# Detection of epileptiform activity in EEG signals based on time-frequency and non-linear analysis

**DOI:** 10.3389/fncom.2015.00038

**Published:** 2015-03-24

**Authors:** Dragoljub Gajic, Zeljko Djurovic, Jovan Gligorijevic, Stefano Di Gennaro, Ivana Savic-Gajic

**Affiliations:** ^1^Department of Signals and Systems, School of Electrical Engineering, University of BelgradeBelgrade, Serbia; ^2^Center of Excellence DEWS, University of L'AquilaL'Aquila, Italy; ^3^Faculty of Engineering, University of KragujevacKragujevac, Serbia; ^4^Faculty of Technology, University of NisLeskovac, Serbia

**Keywords:** seizure detection, epileptiform activity, non-linear analysis, scatter matrices, quadratic classifiers

## Abstract

We present a new technique for detection of epileptiform activity in EEG signals. After preprocessing of EEG signals we extract representative features in time, frequency and time-frequency domain as well as using non-linear analysis. The features are extracted in a few frequency sub-bands of clinical interest since these sub-bands showed much better discriminatory characteristics compared with the whole frequency band. Then we optimally reduce the dimension of feature space to two using scatter matrices. A decision about the presence of epileptiform activity in EEG signals is made by quadratic classifiers designed in the reduced two-dimensional feature space. The accuracy of the technique was tested on three sets of electroencephalographic (EEG) signals recorded at the University Hospital Bonn: surface EEG signals from healthy volunteers, intracranial EEG signals from the epilepsy patients during the seizure free interval from within the seizure focus and intracranial EEG signals of epileptic seizures also from within the seizure focus. An overall detection accuracy of 98.7% was achieved.

## Introduction

According to the estimations of the World Health Organization around 50 million people worldwide suffer from epilepsy as the most common disorder of the brain activity (World Health Organization, 2012). It is characterized by sudden and recurrent seizures which are the result of an excessive and synchronous electrical discharge of a large number of neurons. Epileptic seizures can be divided by their clinical manifestation into two main classes, partial and generalized (Tzallas et al., 2007). Partial or focal epileptic seizures involve only a circumscribed region of the brain (epileptic focus) and remain restricted to this region while generalized epileptic seizures involve almost the entire brain. Both classes of epileptic seizures can occur at all ages. An epileptiform activity in EEG signals including spikes, sharp waves, or spike-and-wave complexes can be evident not only during a seizure (the ictal period) but also a short time before (the preictal period) as well as between seizures (the interictal period). Consequently, EEG signals have been the most utilized in clinical assessments of the brain state including both prediction and detection of epileptic seizures (Waterhouse, 2003; Casson et al., 2010). However, the detection of epileptiform activity in EEG signals by visual scanning of EEG recordings usually collected over a few days is a tedious and time-consuming process. In addition, it requires a team of experts to analyze the entire length of the EEG recordings in order to detect epileptiform activity. A reliable technique for detection of epileptiform activity in EEG signals would ensure an objective and facilitating treatment of patients and thus improve the diagnosis of epilepsy. Furthermore, it would also enable an automated prediction and/or detection of epileptic seizures in real time by a system to be implanted in head of epileptic patients (Jerger et al., 2001). Such a system would significantly improve quality of life of people suffering from epilepsy. Most of the techniques for automated detection of epileptiform activity that have emerged in recent years consist of two key successive steps: extraction of features from EEG signals and then classification of the extracted features for detection of epileptiform activity.

The feature extraction, as the first step, has a direct influence on both precision and complexity of the entire technique. Most common statistical features in time domain, such as the mean, the variance, the coefficient of variation and the total variation, by themselves are not sufficient for a reliable detection of epileptiform activity, and thus are mostly used as statistical measures for features in other domains. The variance and the total variation are considered to have better discriminatory capabilities than the mean, since they are able to detect magnitude of change in a signal over time. Even though we can note a certain periodicity and synchronization between EEG signals from different electrodes, neither the autocorrelation nor the cross-correlation have proved to be reliable features for detection of epileptiform activity. This is especially true in the case of the cortical EEG where the recording electrodes are so close to each other that the synchronization could be noted even when there was no seizure. However, in the literature we can still find several applications of these two features (Niederhauser et al., 2003; Jerger et al., 2005).

Unlike the previous features, the spectral features of EEG signals obtained through the Fourier transform have found wide applications in the field (Polat and Gunes, 2007; Mousavi et al., 2008). Namely, all the research carried out to date clearly indicates that it is much better to identify and extract the features of interest in frequency domain than in time domain, even though the both domains contain identical information. The analysis in time-frequency domain gives even better results considering that it contains, in addition to frequency, also the temporal component of signal which is lost during the Fourier transform. The literature mainly contains techniques based on wavelet transform (Subasi, 2007a,b; Wang et al., 2011; Gajić et al., 2014) which has also been used in the research related to other brain disorders, such as schizophrenia (Hazarika et al., 1997) and Alzheimer's disease (Adeli and Ghosh-Dastidar, 2010). The detection of epileptiform activity based on non-linear analysis, i.e., extraction of the correlational dimension and the Lyapunov exponents as non-linear features can also be noted in some research studies (Iasemidis et al., 2003; Srinivasan et al., 2007; Adeli and Ghosh-Dastidar, 2010).

A precise classification as the second key step directly depends on the previously extracted features. That is, there is no classifier which could in any way make up for the shortcomings which are consequence of the information lost during the feature extraction. Like in the case of the feature extraction, we can come across a very wide range of classifiers starting from the most simple ones with thresholds (Altunay et al., 2010) or rule-based (Gotman, 1999), to linear classifiers (Liang et al., 2010; Iscan et al., 2011) and all the way to those more complex ones based on fuzzy logic and artificial neural networks (Gajić, 2007; Subasi, 2007a; Tzallas et al., 2007). We can also note the use of other techniques for classification based on k nearest neighbors (Guo et al., 2011; Orhan et al., 2011), decision trees (Tzallas et al., 2009), expert models (Ubeyli, 2007; Ubeyli and Guler, 2007) as well as Bayes classifiers (Tzallas et al., 2009; Iscan et al., 2011). Considering that the feature extraction as a process of higher priority can be computationally very demanding it is always more desirable to use simpler classifiers so that the entire decision-making system could ideally work in real time.

In this paper we present an automated technique for detection of epileptiform activity in EEG signals. In contrast with the existing techniques which are mainly based on features from one domain of interest, our new technique optimally integrates features from a few domains and frequency sub-bands of clinical interest in order to increase its robustness and accuracy. We extract features in both time and frequency domain as well as time-frequency domain using discrete wavelet transform which has already been recognized as a very good linear technique for analysis of non-stationary signals such as EEG signals. In addition, by non-linear analysis we extract the correlation dimension and the largest Lyapunov exponent as much better measures of EEG signal non-linearity which is only approximated by other linear techniques such as fast Fourier transform (FFT) and discrete wavelet transform (DWT). After the feature extraction we optimally reduce the feature space dimension to two using scatter matrices and then perform classification in the reduced feature space by quadratic classifiers which have already been known as very robust solutions for classification of random feature vectors.

## Materials and methods

### Materials

**Figure 1:**
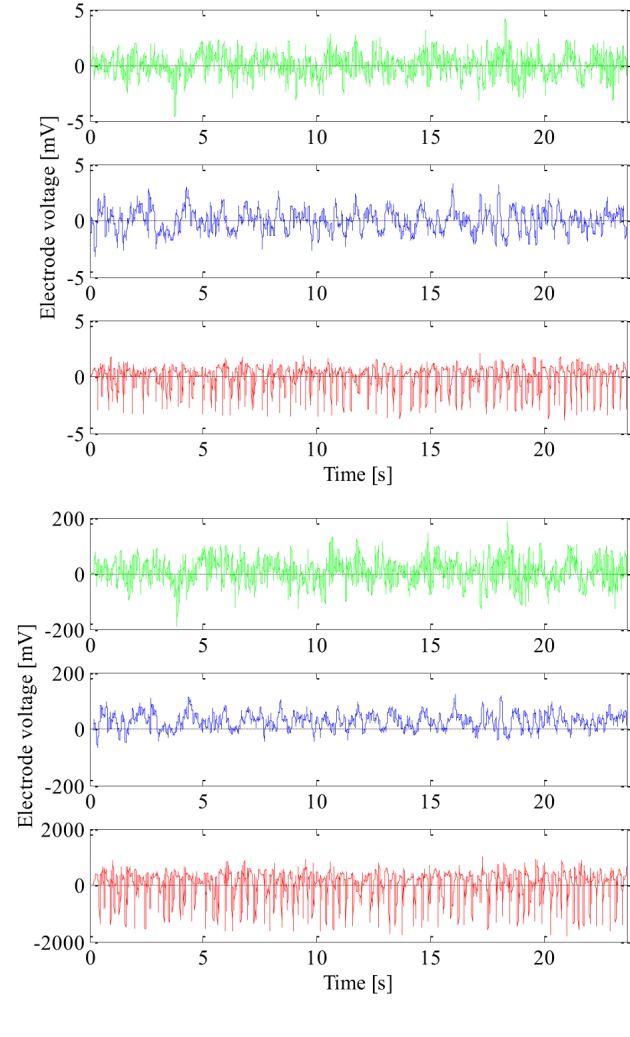
**Non-normalized (lower) and normalized (upper) epileptic (in red) and non-epileptic (unhealthy in blue and healthy tissue in green) EEG signals**.

The EEG signals used to design and test the new technique were recorded at the University Hospital Bonn, Germany with the same 128-channel amplifier system (Andrzejak et al., 2001). After 12 bit analog-to-digital conversion the EEG signals were saved in a data acquisition system at a sampling rate of 173.61 Hz. The amplifier range was adjusted well so that the recordings could be made with 12 bits. The recorded EEG signals were further passed through a low pass filter with the finite impulse response and bandwidth of 0–60 Hz. The frequencies higher than 60 Hz mostly present noise and are a very small part of the signal total energy in the frequency band up to 86.8 Hz saved by the acquisition system. We used 100 segments of epileptic and 200 segments of non-epileptic EEG signals to design and test our new technique. The epileptic EEG signals were recorded using cortical electrodes from 5 epileptic patients during seizure from within the seizure focus, i.e., the region of unhealthy brain tissue that was later removed by surgery. The first 100 segments of non-epileptic EEG signals were also recorded using cortical electrodes from the same epileptic patients and the same unhealthy brain tissue but during seizure-free interval. The remaining 100 segments of non-epileptic EEG signals were recorded using scalp electrodes from 5 healthy volunteers and of course their healthy brain tissue. So, there was a total of three groups with 100 segments of the EEG signals. All the segments have duration of 4096 samples, i.e., 23.6 s, and were additionally tested on the weak stationarity (Andrzejak et al., 2001) in order to perform non-linear analysis. Since the EEG signals were recorded from different patients and with different electrodes, all extracted EEG signal segments were also additionally normalized in order to have the same zero mean and unit variance as shown in Figure 1. In this way, we wanted to design a detection technique that is not dependent on patient and the EEG recording system either.

### Methods

**Figure 2:**
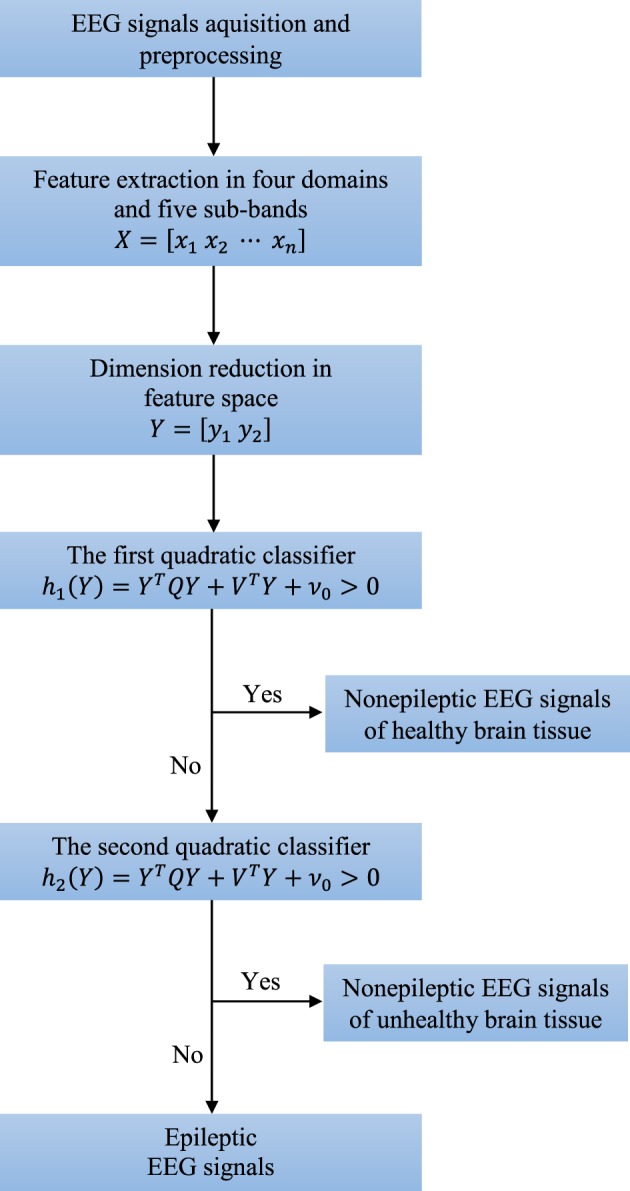
**Structure of the new technique consisting of four key steps: preprocessing, feature extraction, dimension reduction, and classification**.

There are five broad sub-bands of the EEG signal which are generally of clinical interest: delta (0–4 Hz), theta (4–8 Hz), alpha (8–16 Hz), beta (16–32 Hz), and gamma waves (32–64 Hz). Higher frequencies are often more common in abnormal brain states such as epilepsy, i.e., there is a shift of EEG signal energy from lower to higher frequency bands before and during a seizure (Gajić et al., 2014). These five frequency sub-bands provide more accurate information about neuronal activities underlying the problem. Consequently, some changes in the EEG signal, which are not so obvious in the original full-spectrum signal, can be amplified when each sub-band is considered independently. Thus, we extract features from each sub-band separately and also in time, frequency and time-frequency domain as well as by non-linear analysis. After the feature extraction we reduce dimension of the feature space to two. Finally, two quadratic classifiers able to separate all three groups of the EEG signals from each other are designed. The entire structure of the technique is shown in Figure 2.

#### Time-frequency domain analysis

Since the segments of the EEG signals have already been normalized and all have zero mean and unit variance, additional extraction of these two features as well as coefficient of variation as function of mean value and variance, does not make any sense. However, we extracted the total variation as another measure of signal variability in the time domain even after normalization since it counts number of signal sign changes or signal polarity. In the case of a signal segment *x*[*n*] of N samples, i.e., *n* = 1, 2 ··· *N*, the total variation is given by:

(1) $$v_{x}=\frac{1}{N-1}\frac{\sum_{n=2}^{N}\left|x\left[n\right]-x[n-1]\right|}{\left(max_{x}-min_{x}\right)}$$

where the signal is essentially normalized by the difference between its maximum and minimum values in the segment of interest. Obviously, the value of the total variation is located in the range between 1/(*N* − 1) for slower signals and 1 for signals with very high and frequent changes.

EEG signals, as the outcome of events with different repetition periods, contain signals whose different frequencies cannot be identified in the time domain, since all these signals are shown together. Thus, signal transformation from the time domain to the frequency domain is necessary, which in the case of a signal segment *x*[*n*] of *N* samples is achieved using the fast Fourier transform (FFT) defined by:

(2) $$fft\left[\omega \right]=\sum_{n=1}^{N}x[n]e^{-i\omega n},\;\omega =\frac{2\pi m}{N},\;0\leq m\leq N-1$$

where ω = 2π*f*/*f_s_* represents the angular frequency discretized in *N* samples (Proakis and Manolakis, 1996). In order to avoid discontinuities between the end and beginning of the segments and thus spurious spectral frequency components the beginning of each segment was chosen in such a way that the amplitude difference of the last and first data points was within the range of amplitude differences of consecutive data points, and the slopes at the end and beginning of each segment had the same sign. This procedure reduces edge effects that result in spectral leakage in the FFT spectrum. In order to further minimize spectral leakage windowing of signal segments by the Hamming window (the sum of a rectangle and a Hanning window) is used before application of the FFT. Considering the fact that by transforming the signal into the frequency domain we do not lose any original information from the time domain, the signal can completely be reconstructed using the inverse Fourier transform by:

(3) $$x[n]=\frac{1}{N}\sum_{\omega =0}^{2\pi (N-1)/N}fft[\omega ]e^{i\omega n},\;1\leq n\leq N$$

Clearly, the longer the segment *x*[*n*], i.e., the larger *N*, the greater the frequency resolution.

Power spectral density is also one of the most important features of the signal in the frequency domain and represents the contribution of each individual frequency component to the power of the whole signal segment *x*[*n*]. In practice, power spectral density is usually estimated using the coefficients of the fast Fourier transform, i.e., the periodogram (Welch, 1967) given by:

(4) $$per\left[\omega \right]=\frac{1}{N}{\left|fft[\omega ]\right|}^{2}$$

which is an unbiased and inconsistent estimator. Thus, with the increase in the length of the signal segment, the mean of the estimation tends toward the actual value of power spectral density, which is actually an advantage, unlike variance estimation, which is not reduced, i.e., which does not have a tendency toward zero with the increase in segment length. A periodogram can be further normalized by the total signal power, i.e.,:

(5) $$per_{norm}\left[\omega \right]=\frac{1}{N}{\left|fft\left[\omega \right]\right|}^{2}/\sum_{\omega =0}^{2\pi (N-1)/N}per\left[\omega \right]$$

where we obtain the relative contribution of each frequency component to the total power of the signal. If the original signal segment *x*[*n*] is further divided into P sub-segments of the *N*/*P* samples, the periodogram can be calculated as follows:

(6) $$per\left[\omega \right]=\frac{1}{P}\sum_{p=0}^{P-1}\frac{P}{N}{\left|fft_{p}[\omega ]\right|}^{2}$$

where *fft_p_*[ω] is the fast Fourier transform of each of the sub-segments of the *N*/*P* sample. In this way, the periodogram is actually an averaged one with a smaller variance, but clearly with a lower resolution in the frequency domain. Based on the periodogram we extracted relative power of all five previously mentioned sub-bands, i.e., delta (0–4 Hz), theta (4–8 Hz), alpha (8–12 Hz), beta (12–30 Hz), and gamma (30–60 Hz), as features of interest in frequency domain.

By analyzing the EEG signals solely in the time domain, extracted features do not contain any information on frequencies, which are, as we will later show, also very important for the proper detection of epileptic EEG signals. On the other hand, by transforming the signals from the time into the frequency domain, any information on time is completely lost, except of course in the case of sequential application on sufficiently short and stationary sub-segments, which also has its disadvantage in terms of the correct choice of the length of these sub-segments which would enable the simultaneous achievement of the desired resolution in both domains. In addition, once selected, the sub-segment length, i.e., the resolution in the time domain, remains fixed throughout the entire frequency bands and cannot be adjusted to the dominant signal frequencies at a specific time. Signal processing using wavelets very accurately resolves this deficiency and results in sufficient information on non-stationary signals, both in the time and frequency domain. We are already familiar with the fact that a signal can be presented as a linear combination of its basic functions. A unit impulse function whose power is limited and whose mean differs from zero is the basic function of the signal in the time domain, whereas in the frequency domain, this role is assigned to the sinusoidal function that has infinite power, and a zero mean. In the time-frequency domain, the basic function is the wavelet, which is actually a function of limited power, i.e., duration, and a zero mean (Rao and Bopardikar, 1998), and for which the following is valid:

(7) $$\sum_{n=-\infty }^{\infty }{\left|\psi [n]\right|}^{2}<\infty ,\sum_{n=-\infty }^{\infty }\psi \left[n\right]=0.$$

The wavelet that is moved, or translated, in time for b samples and scaled by the so-called dilation parameter *a* is given by:

(8) $$\psi_{ab}[n]=\frac{1}{\sqrt{a}}\psi \left[\frac{n-b}{a}\right].$$

By changing the dilation parameter, the basic wavelet (*a* = 1) changes its width, that is, it spreads (*a* > 1) and contracts (0 ≤ *a* < 1) in the time domain. In the analysis of non-stationary signals, the possibility of changing the width of the wavelet represents a significant advantage of this analysis technique, considering the fact that wider wavelets can be used to extract slower changes, i.e., lower signal frequencies, and narrower wavelets can be used to extract faster changes, i.e., higher frequencies. Following the selection of the values of parameters *a* and *b* it is possible to transform segments of the signal *x*[*k*] of *N* samples, that is, to calculate the wavelet transform coefficients in the following way:

(9) $$w_{ab}[n]=\sum_{\tau =1}^{N}x[\tau ]\psi_{ab}\left[n-\tau \right],\;1\leq n\leq N$$

Thus, what is actually being extracted from the signal are only those frequencies that are within the wavelet frequency band ψ_*ab*_[*n*], i.e., the signals are filtrated by the wavelet ψ_*ab*_[*n*]. As previously indicated, based on the coefficients obtained in this way, the original signal can be reconstructed using an inverse wavelet transform. Of course, if necessary, it is possible to also independently reconstruct the part of the signal which is filtered, as well as the part that was rejected by the wavelet ψ_*ab*_[*n*] on the basis of the so-called detail coefficients and approximation coefficients respectively, which are of course a function of the transformation coefficients ψ_*ab*_[*n*].

Parameters *a* and *b* can continuously change, which is not so practical especially bearing in mind that the signal can be completely and accurately transformed and reconstructed by using a smaller and finite number of wavelets, that is, by using a limited number of discrete values of parameters *a* and *b*, which is also known as the discrete wavelet transform (DWT). In this case, parameters *a* and *b* are the powers of 2, which gives us the dyadic orthogonal wavelet network with frequency bands which do not overlap each other. The dilation parameter a, as the power of 2, at each subsequent higher level of transformation, doubles in value in comparison to the value from the previous level, which means that the wavelet becomes twice as wide in the time domain, and has a frequency band that is half as narrow and twice as low. This actually decreases the resolution of the transformed signal in the time domain two-fold, increasing it twice as much in the frequency domain. Thus, the signal frequency band from the previous level is split into two halves at every next level, into a higher band which contains higher frequencies and describes the finer changes, or details, and a lower band that contains lower frequencies and actually represents an approximation of the signal from the previous level. This technique is also known as wavelet decomposition of the signal.

Before the application of DWT, it is necessary to choose the type of the basic wavelet as well as the number of levels into which the signal will be decompose. After analysis of several types of the basic wavelets, the fourth-order Daubechies wavelet (Rao and Bopardikar, 1998) was selected for further analysis within this work since it has good localizing properties both in the time and frequency domains (Kalayci and Özdamar, 1995; Petrosian et al., 2000) Due to its shape and smoothing feature this type of the basic wavelet has already shown good capabilities in the field of EEG signal processing. The discrete wavelet decomposition was performed at four levels that resulted into five sub-bands of clinical interest. The standard deviation and the average relative power of the DWT coefficients in each of the sub-bands were extracted as representative features in time-frequency domain.

#### Non-linear analysis

EEG signals, as the result of the activities of an extremely complex and non-linear system, in addition to the fairly well-known and previously described linear techniques, can also be analyzed using some of the non-linear techniques. By using linear techniques, any non-linearity that can be found in the signal is only approximated, which can result in the loss of certain pieces of potentially relevant information. If that is the case, the use of non-linear techniques is preferred since they are more reliable for non-linear analyses, despite the fact that they imply weak signal stationarity (Varsavsky et al., 2011), and the fact that they need somewhat longer segments, which leads to their being computationally more demanding than linear techniques.

Let *x*[*n*] again represent the signal segment which is to be analyzed, where *n* = 1 ··· *N*. Also, let *m* denote the lag for which we can define two new sub-segments *x*[*n*], the first *x_k_* containing samples starting from *k* up to *N* − *m* and the second *x*_*k* + *m*_ with samples starting from *k* + *m* to *N*. Both of these sub-segments contain *N* − *k* − *m* + 1 samples and can be represented opposite one another in the phase space with a lag *m* and the so-called embedding dimension 2. In case of three sub-segments: *x*_*k* + 2*m*_, *x*_*k* + *m*_ and *x_k_*, the embedding dimension of the phase space would be 3. The lagged phase space provides a completely different view of signal evolution in time, where we can note that the signal gravitates to a certain part of the phase space, known as the attractor. With the aim of constructing lagged phase space, i.e., the signal attractor, it is necessary to previously define the values of the lag and the embedding dimension, which although significantly smaller than the real dimension of the non-linear system space, provides an approximation of the signal complexity and non-linearity (Andrzejak et al., 2001). The lag *m* should be large enough so that these sub-segments would overlap as little as possible, that is, share as little mutual information as possible, but at the same time sufficiently small so that the sub-segments could be long enough for any further useful analysis. An optimal lag is obtained by determining the mutual information coefficient the sub-segments for different values of the lag *m*. The mutual information coefficient is defined by Williams (1997):

(10) $$info_{m}=\sum_{i=1}^{N_{s}}\sum_{j=1}^{N_{s}}p\left(x_{k}\left[i\right],x_{k+m}[j]\right)log_{2}\frac{p\left(x_{k}[i],x_{k+m}[j]\right)}{p\left(x_{k}\left[i\right]\right)p\left(x_{k+m}[j]\right)}$$

where *N_s_* represents the number of areas in which the signal is discretized based on the amplitude and *p* is the corresponding probability that the sub-segment belongs to a certain area. The first local minimum shown in the graph representing the dependence of the mutual information coefficient on lag determines the optimal lag *m_o_*.

After determining the optimal lag, the minimum embedding dimension of the lagged phase space is estimated using Cao's technique (Cao, 1997). In the phase space with a lag *m_o_* and embedding dimension *d*, the original segment is represented by its phase portraits, which all together make up the attractor defined by the following points in the lagged phase space:

(11) $$y_{d}[i]=\left[x[i]\;x\left[i+m_{o}\right]\;\cdots \;x\left[i+m_{o}(d-1)\right]\right]$$

where *i* = 1, 2, ···, *N* − *m_o_*(*d* − 1). According to the technique developed by Cao, if *d* is the right dimension, then the two points are also close to each other in phase space dimension *d*, as well as in the phase space of dimension *d* + 1 and are referred to as real neighbors (Cao, 1997). Dimension increases gradually until the number of false neighbors reaches zero, that is, until the Cao's embedding function defined by:

(12) $$e_{d}=\frac{1}{N-m_{o}d}\sum_{i=1}^{N-m_{o}d}\frac{\left\|y_{d+1}[i]-y_{d+1}[n_{i,\;d}]\right\|}{\left\|y_{d}[i]-y_{d}[n_{i,\;d}]\right\|}$$

becomes constant, where *i* = 1, 2, ···, *N* − *m_o_d* and *y_d_*[*n*_*i*, *d*_] represents the nearest neighbor of *y_d_*[*i*] in the *d*-dimensional phase space with a lag *m_o_*. In fact, the minimum embedding dimension *d_min_* is determined when the ratio between the *e*_*d* + 1_/*e_d_* approaches the value of 1. Since this ratio may approach 1 in some other cases, e.g., for completely random signals, an additional check is also carried out where the Cao's embedding function is redefined and given by:

(13) $$e_{d}^{*}=\frac{1}{N-m_{o}d}\sum_{i=1}^{N-m_{o}d}\left|x\left[i+m_{o}d\right]-x\left[n_{i,\;d}+m_{o}d\right]\right|$$

where *x*[*n*_*i, d*_ + *m_o_d*] is the nearest neighbor of *x*[*i* + *m_od_*]. The constant value of the ratio e^*^_d + 1_/e^*^_d_ for different values of the embedding dimension indicates that we are dealing with a random signal. The signal is not random, i.e., it is deterministic if this ratio differs from 1 for at least one value of the embedding dimension, which in that case is also the minimum value.

The correlation dimension is a measure of the complexity of the signal attractor in the lagged phase space. This dimension, unlike most others better known dimensions, may have a fractional value and could thus characterize the dimension, that is, the complexity of the attractors with more precision than the embedding dimension; however, it is always less than or equal to the embedding dimension.

Let *C*_ε_ be the correlational sum of the signal segment with *N* samples within the radius ε in its phase space with a lag *m_o_* and minimum embedding dimension *d_min_*, i.e., *M* = *N* − *m_o_d_min_* points *y_d_min__* given by Williams (1997):

(14) $$C_{\varepsilon }=\underset{M\to \infty }{lim}\frac{1}{M^{2}}\sum_{i=1}^{M}\sum_{j=1}^{M}H(\varepsilon -\left\|y_{d_{min}}[i]-y_{d_{min}}[j]\right\|)$$

where *H* is the Heaviside step function that results in 1 if *y_d_min__*[*j*] is within the radius ε of *y_d_min__*[*i*], i.e.,:

(15) $$\varepsilon -\left\|y_{d_{min}}[i]-y_{d_{min}}[j]\right\|>0$$

otherwise it is 0. The correlation dimension *d_corr_* is the approximated slope of the natural logarithm of the correlation sum as a function of ε. Given that the total number of possible distances between two points in a lagged phase space equals *M*(*M* − 1)/2, the correlation dimension could directly be obtained by the Takens estimator (Takens, 1981; Cao, 1997) using:

(16) $$d_{corr}=-\left[\text{​​}\frac{2}{M\left(M-1\right)}\sum_{i=1}^{M}\sum_{j=1}^{M}log\left(\text{​}\frac{\left\|y_{d_{min}}\left[i\right]-y_{d_{min}}\left[j\right]\right\|}{\varepsilon }\text{​}\right)\text{​​}\right]$$

The largest Lyapunov exponent λ_*max*_ represents a measure of both chaotic behavior of the attractor and the divergence of the trajectories in phase space, i.e., the predictability of the signal. Attractor divergence is the distance between two closely positioned points in a phase space after a certain period of time of *k* samples, which is also known as the prediction length. Based on chaos theory, i.e., the so-called butterfly effect, two points close in the phase space of a chaotic system may have completely different trajectories. Thus, the divergence of the trajectories implies a chaotic system, and vice versa. The Lyapunov exponent actually characterizes the exponential growth of that divergence. The number of Lyapunov exponents is equal to the embedding dimension, and each of these Lyapunov exponents represents the rate of a contracting (λ <0) or expanding attractor (λ >0) in a certain direction of the phase space. In the case of a chaotic system, the trajectories must diverge in at least one dimension, which means that at least one Lyapunov exponent must be greater than zero, when it is, at the same time, the largest Lyapunov exponent. If several Lyapunov exponents are positive, then the largest among them indicates the direction of the maximum expansion of the attractor and its chaotic behavior. The mean of the trajectory divergence after *k* samples and a sampling period *T_s_* can be calculated by the Wolf's technique (Wolf et al., 1985; Rosenstein et al., 1993) using:

(17) $$d_{T}=\frac{1}{\left(M-k\right)}\sum_{i=1}^{M-k}\frac{\left\|y_{d_{min}}[i+k]-y_{d_{min}}[n_{i}+k]\right\|}{\left\|y_{d_{min}}\left[i\right]-y_{d_{min}}[n_{i}]\right\|}$$

where *y_d_min__*[*i*] and *y_d_min__*[*n_i_*] represent two close points on different trajectories in the phase space. The largest Lyapunov exponent λ_*max*_ is in this case an approximation of the slope of the natural logarithmic trajectory divergence as a function of the number of samples *k*, i.e., *d_T_* = *d*_0_*e*^*kT*_*s*_λ_*max*_^ where *d*_0_ stands for the initial divergence. In addition, there is another very similar more practical technique for the evaluation of the largest Lyapunov exponent proposed by Sato et al. where we first calculate the prediction error for several different values of the number of samples *k* using:

(18) $$p_{k}=\frac{1}{\left(M-k\right)}\sum_{i=1}^{M-k}log_{2}\frac{\left\|y_{d_{min}}\left[i+k\right]-y_{d_{min}}[n_{i}+k]\right\|}{\left\|y_{d_{min}}\left[i\right]-y_{d_{min}}[n_{i}]\right\|}$$

after which the λ_*max*_ is determined as the slope of the middle and approximately linear part of the prediction error *p_k_* as a function of *kT_s_*.

We extract both the correlation dimension and the largest Lyapunov exponent as features that describe complexity and chaotic behavior of the attractor in the lagged phase space. By choosing the radius ε, the phase space is divided into parts of the dimension ε. While the correlation dimension shows how many points can be found in the surrounding areas of the phase space, the Lyapunov exponent describes the distance between each of the trajectories that terminate in different parts of the phase space but start from the same one. In other words, both of these features give us an idea of how complex and predictable EEG signal is, which, of course, they both interpret and quantify in their own characteristic way.

#### Dimension reduction in feature space

Let an *n*-dimensional random vector *X* be transformed through the application of a certain linear transformation into an *n*-dimensional random vector *Y* = *A^T^X* where *A* is the transformational square matrix of the dimension *n*. Then the mean vector and the covariance matrix of the random vector *Y* are *M_Y_* = *A^T^M_X_* and Σ_*Y*_ = *A^T^*Σ_*X*_*A*. Based on that, the distance function is:

(19) $$\begin{array}{l} d_{Y}^{2}(Y)={\left(Y-M_{Y}\right)}^{T}\Sigma_{Y}^{-1}(Y-M_{Y})={\left(X-M_{X}\right)}^{T}\Sigma_{X}^{-1}\\ \;(X-M_{X})=d_{X}^{2}(X) \end{array}$$

that is, the distance function does not change with the linear transformation. If we were to perform the translation of the coordinate system for the mean vector *M_X_* we would obtain the random vector *Z* = *X* − *M_X_* whose mean vector is zero and its covariance matrix is the same as Σ_*X*_. If we wanted to determine the random vector Z which maximizes the distance function *d*^2^_*Z*_(*Z*) = *Z^T^*Σ^−1^*Z* under the condition that *Z^T^Z* = 1, it is necessary to minimize the following criterion:

(20) $$J=Z^{T}\Sigma^{-1}Z-\mu \left(Z^{T}Z-1\right)$$

where μ is the Lagrange multiplier. By using a partial derivate ∂*J*/∂*Z* and by equating it with zero, we obtain the following:

(21) $$\partial J/\partial Z=2\Sigma^{-1}Z-2\mu Z\;\Rightarrow \Sigma Z=\lambda Z$$

where λ = 1/μ. With the aim of obtaining a non-zero solution which satisfies the equation:

(22) ΣZ=λZ ⇔ (Σ−λI)Z=0

it is further necessary to find such a parameter λ which satisfies the following so-called characteristic equation of a matrix Σ:

(23) |Σ−λI|=0

Every λ which satisfies this characteristic equation is known as eigenvalue of the matrix Σ while the vector *Z* related to specific eigenvalue is known as an eigenvector. When Σ is a symmetric *n* × *n* matrix, then there are *n* real eigenvalues λ_1_, λ_2_, …, λ_*n*_ and *n* real eigenvectors Φ_1_, Φ_2_, …, Φ_*n*_ which are mutually orthogonal and for which Σ Φ = Φ Λ and Φ^*T*^Φ = *I* where Φ = [Φ_1_ Φ_2_ ··· Φ_*n*_] is the square matrix of the eigenvectors, Λ the diagonal matrix of the eigenvalues:

(24) $$\Lambda =\left[\begin{array}{ccc} \lambda_{1} & \cdots & 0\\ \vdots & \ddots & \vdots \\ 0 & \cdots & \lambda_{n} \end{array}\right]$$

while *I* is the identity matrix.

If the matrix Φ is used as a transformation matrix during the linear transformation *Y* = Φ*^T^X*, then the covariance matrix of the random vector *Y* will be Σ_*Y*_ = Φ^*T*^Σ_*X*_Φ = Λ. This kind of transformation is orthonormal since for the transformation matrix Φ holds Φ^*T*^Φ = *I*. In addition, during all these orthonormal transformations, the Euclidean distance does not change, that is ||*Y*||^2^ = *Y^T^Y* = *X^T^*Φ^*T*^Φ*X* = *X^T^X* = ||*X*||^2^.

Let *X* be an *n*-dimensional random vector of the extracted features which could be represented using n linear independent vectors in the following way:

(25) $$X=\sum_{i=1}^{n}y_{i}\Phi_{i}=\Phi Y$$

where Φ = [Φ_1_ Φ_2_ ··· Φ_*n*_] and *Y* = [*y*_1_ *y*_2_ ··· *y_n_*] that is Φ_*i*_ are the basis vectors of the new *n*-dimensional space, and the new coordinates *y_i_* are the scalar products of the basis vectors Φ_*i*_ and the random vector *X*. Assuming that the columns of the matrix Φ or in other words the basis vectors Φ_*i*_ are orthogonal, the coordinates of the random vector *X* in the new space can be obtained in the following way:

(26) $$y_{i}=\Phi_{i}^{T}X.$$

**Figure 3:**
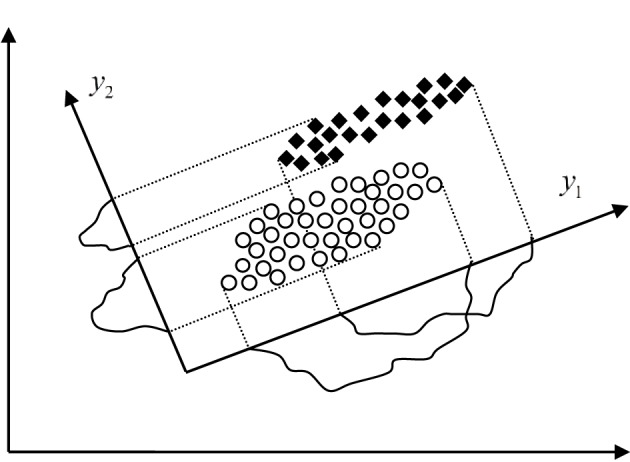
**Different approaches to dimension reduction in feature space, the KL expansion technique which rejects the feature y_2_ and the technique based on scatter matrices which rejects the feature y_1_**.

Thus, Y represents a mapped random vector and the orthonormal transformation of the original random vector *X*. The random vector *X* approximated using only the *m* (*m* < *n*) basis vectors, i.e., the mapped features, could be represented in the following way:

(27) $$\hat{X}(m)=\sum_{i=1}^{m}y_{i}\Phi_{i}+\sum_{i=m+1}^{n}b_{i}\Phi_{i}$$

where the approximation error becomes:

(28) $$\Delta X(m)=X-\hat{X}(m)=\sum_{i=m+1}^{n}(y_{i}-b_{i})\Phi_{i}$$

and the mean squared error:

(29) $${\bar{\varepsilon }}^{2}(m)=E\left\{{\left\|\Delta X(m)\right\|}^{2}\right\}=\sum_{i=m+1}^{n}E\left\{{\left(y_{i}-b_{i}\right)}^{2}\right\}$$

has its own minimal value for *b_i_* = *E*{*y_i_*} = Φ*^T^_i_E*{*X*}. The optimal mean squared error can then be presented in the following form:

(30) $$\begin{array}{l} {\bar{\varepsilon }}_{opt}^{2}(m)=\sum_{i=m+1}^{n}E\left\{{\left(y_{i}-E\left\{y_{i}\right\}\right)}^{2}\right\}\\ \;=\sum_{i=m+1}^{n}\Phi_{i}^{T}E\left\{(X-E\left\{X\right\}){\left(X-E\left\{X\right\}\right)}^{T}\right\}\Phi_{i}\\ \;=\sum_{i=m+1}^{n}\Phi_{i}^{T}\Sigma_{X}\Phi_{i}=\;\sum_{i=m+1}^{n}\lambda_{i} \end{array}$$

where Σ_*X*_ is the covariance matrix of the random vector *X* and λ_*i*_ are its eigenvalues. Thus, the minimal mean squared error of approximation is also equal to the sum of the eigenvalues of the leftout coordinates, which actually means that we should leave out coordinates with the smallest eigenvalues. The mapping of the random vector *X* into the space made up by the eigenvectors of its covariance matrix Σ_*X*_ is known as the Karhunen-Loeve (KL) expansion. When reducing the dimension of the feature space using the KL expansion technique we should bear in mind that the performance of each feature is characterized by its eigenvalue. Thus, by rejecting features we should first reject those with the smallest eigenvalue, i.e., with the smallest variance in the new feature space. For example, in the case of dimension reduction from two to one shown in Figure 3 the feature *y*_2_ would be rejected as less informative even though it has better discriminatory potential than *y*_1_. Also the coordinates *y_i_* are mutually uncorrelated considering that the covariance matrix of the random vector *Y* is diagonal, i.e.,:

(31) $$\Sigma_{Y}=\Phi^{T}\Sigma_{X}\Phi =\Lambda =diag\left\{\lambda_{1}\lambda_{2}\cdots \lambda_{\text{n}}\right\}.$$

Unlike the previously outlined method, the reduction of dimension based on scatter matrices (Fukunaga, 1990; Djurovic, 2006) is of special significance for the new detection technique since it takes into consideration the very purpose of the reduction, that is, the classification of the random vectors. Let *L* be the number of classes which should be classified and *M_i_* and Σ_*i*_, *i* = 1 ··· *L* the mean vectors and the covariance matrices of these classes, respectively. Then the within-class scatter matrix can be defined by:

(32) $$S_{W}=\sum_{i=1}^{L}P_{i}E\left\{\left(X-M_{i}\right){\left(X-M_{i}\right)}^{T}/\omega_{i}\right\}=\sum_{i=1}^{L}P_{i}\Sigma_{i}$$

and the between-class scatter matrix as:

(33) $$S_{B}=\sum_{i=1}^{L}P_{i}\left(M_{i}-M_{0}\right){\left(M_{i}-M_{0}\right)}^{T}$$

where *M*_0_ is the joint vector of mathematical expectation for all the classes together, that is:

(34) $$M_{0}=E\left\{X\right\}=\sum_{i=1}^{L}P_{i}M_{i}.$$

In addition the mixed scatter matrix can be defined by:

(35) $$S_{M}=E\left\{\left(X-M_{0}\right){\left(X-M_{0}\right)}^{T}\right\}=S_{W}+S_{B}.$$

Then the problem of dimension reduction is reduced to the identification of the *n* × *m* transformation matrix *A* which maps the random vector *X* of dimension *n* onto the random vector *Y* = *A^T^X* of dimension *m* and at the same time maximizes the criteria *J* = *tr*(*S*^−1^_*W*_*S_B_*). This criteria is invariant to non-singular linear transformations and results into transformation matrix that takes the following form:

(36) $$A=\left[\Psi_{1}\;\Psi_{2}\;\cdots \;\Psi_{m}\right]$$

where Ψ_*i*_, *i* = 1, …, *m* are the eigenvectors of the matrix *S*^−1^_2_*S*_1_ which correspond to the greatest eigenvalues, i.e., (*S*^−1^_*W*_*S_B_*)Ψ_*i*_ = λ_*i*_Ψ_*i*_, *i* = 1, …, *n*, λ_1_ ≥ λ_2_ ≥ ··· ≥ λ_*n*_. Dimension reduction based on scatter matrices applied to the case shown in Figure 3 would result into selection of the feature *y*_2_ that is much better choice than the feature *y*_1_ selected by the KL expansion technique, of course in terms of more accurate classification.

#### Design of quadratic classifiers

Quadratic classifiers are already known to be very good robust solutions to the problems of classification of random vectors whose statistical features are either unknown or change over time. Additionally, quadratic classifiers allow visual insight into the classification results. We design a piecewise quadratic classifier for detection of epileptiform activity, i.e., two quadratic classifiers, able to separate all three classes of the EEG signals of interest as shown in Figure 2. The quadratic classifiers have the same structure defined by the following equation:

(37) $$\begin{array}{l} h\left(Y\right)=Y^{T}QY+V^{T}Y+\nu_{0}\\ \;=\left[y_{1}\;y_{2}\right]\;\left[\begin{array}{cc} q_{11} & q_{12}\\ q_{21} & q_{22} \end{array}\right]\;\left[\begin{array}{c} y_{1}\\ y_{2} \end{array}\right]+\left[\nu_{1}\;\nu_{2}\right]\;\left[\begin{array}{c} y_{1}\\ y_{2} \end{array}\right]+\nu_{0} \end{array}$$

where *y*_1_ and *y*_2_ are two features in the reduced feature space. The matrix *Q*, the vector *V* and scalar ν_0_ are the unknowns which are also need to be determined optimally. The quadratic equation (37) can be represented in a linear form as:

(38) $$h\left(Y\right)=\left[q_{11}\;q_{12}\;q_{22}\;\nu_{1}\;\nu_{2}\right]\;\left[\;\begin{array}{c} y_{1}^{2}\\ 2y_{1}y_{2}\\ \begin{array}{c} y_{2}^{2}\\ y_{1}\\ y_{2} \end{array} \end{array}\right]+\nu_{0}=V_{z}^{T}Z+\nu_{0}.$$

In order to also achieve the largest possible between-class and shortest within-class scattering during the dimension reduction in the feature space, for the optimization criterion we have selected the following function (Fukunaga, 1990):

(39) $$f=\frac{P_{1}\eta_{1}{}^{2}+P_{2}\eta_{2}{}^{2}}{P_{1}\sigma_{1}{}^{2}+P_{2}\sigma_{2}{}^{2}}$$

where *P*_1_ and *P*_2_ are probabilities and

(40) $$\eta_{l}=E\left\{h(Z)/\omega_{l}\right\}=E\left\{V_{z}^{T}Z+\nu_{0}/\omega_{l}\right\}=V_{z}^{T}M_{l}+\nu_{0}$$

(41) $$\sigma_{l}{}^{2}=var\left\{h(Z)/\omega_{l}\right\}=var\left\{V_{z}^{T}Z+\nu_{0}/\omega_{l}\right\}=V_{z}^{T}\Sigma_{l}V_{z}.$$

*M_l_* and Σ_*l*_ are the mean vectors and covariance matrices, respectively, of the random vector *Z* for each of the two classes *l* that need to be classified. By optimizing the function *f*, for the optimal vector *V_z_*, i.e., matrix *Q* and vector *V* from Equation (37), we have:

(42) $$V_{z}=\left[\;\begin{array}{c} q_{11}\\ q_{12}\\ \begin{array}{c} q_{22}\\ \nu_{1}\\ \nu_{2} \end{array} \end{array}\right]={\left[P_{1}\Sigma_{1}+P_{2}\Sigma_{2}\right]}^{-1}\left(M_{2}-M_{1}\right)$$

and for the optimal scalar:

(43) $$\nu_{0}=-V_{z}^{T}\left(P_{1}M_{1}+P_{2}M_{2}\right)$$

which finishes the design of the quadratic classifiers as well as the new technique for detection of epileptiform activity.

Statistical performances such as sensitivity, specificity and accuracy of the designed piecewise quadratic classifier, i.e., the new technique for detection of epileptiform activity, is estimated based on the classification results. The sensitivity is defined as a ratio between the number of correctly classified segments and the total number of the segments for each of the classes separately. The specificity is also calculated for each of these three classes separately and represents the ratio between the number of correctly classified features of the other two classes and the total number of the segments of these two classes. The accuracy is calculated as the ratio between the total number of correctly classified segments and the total number of the segments in all three classes together.

## Results

### Feature extraction

In total 30 features for each of 300 analyzed segments of the EEG signals were extracted. All the features together with their mean values and standard deviations for all three different classes of EEG signals of interest are presented in Table 1. The extracted features refer to the adequate clinical sub-bands since these sub-bands had better discrimination characteristics compared with the whole frequency band between 0 and 60 Hz. The separability index as a measure of the discriminatory potential was also calculated for all the extracted features. In this case, the separability index is the criteria *J* = *tr*(*S*^−1^_*W*_*S_B_*) where *S_W_* and *S_B_* are earlier defined within- and between-class scatter matrices, respectively. Based on these matrices, a higher separability index corresponds to better separability between different classes of the EEG signals. Based on these 30 features, each original segment of the EEG signals from time domain can be presented now by its feature vector *X* = [*x*_1_*x*_2_ ··· *x*_30_]^*T*^, i.e., by the point in the feature space with dimension of 30.

**Table 1:** **Normalized features extracted from different frequency sub-bands**.

**Index**	**Feature**	**Non-epileptic of healthy tissue**		**Non-epileptic of unhealthy tissue**		**Epileptic**		**Separa-bility**
		μ	σ	μ	σ	μ	σ	***J***
*x*_1_	Total variation—delta	0.011	0.002	0.011	0.003	0.019	0.005	1.253
*x*_2_	Total variation—theta	0.027	0.004	0.022	0.006	0.028	0.006	0.300
*x*_3_	Total variation—àlpha	0.044	0.005	0.034	0.011	0.042	0.011	0.215
*x*_4_	Total variation—beta	0.075	0.008	0.057	0.024	0.062	0.023	0.150
*x*_5_	Total variation—gamma	0.149	0.019	0.102	0.047	0.103	0.041	0.335
*x*_6_	Relative power FFT—delta	0.446	0.090	0.628	0.147	0.267	0.220	0.720
*x*_7_	Relative power FFT—theta	0.159	0.049	0.236	0.119	0.390	0.224	0.417
*x*_8_	Relative power FFT—alpha	0.162	0.043	0.086	0.066	0.134	0.057	0.316
*x*_9_	Relative power FFT—beta	0.221	0.075	0.046	0.024	0.205	0.151	0.641
*x*_10_	Relative power FFT—gamma	0.012	0.010	0.004	0.003	0.004	0.005	0.264
*x*_11_	St. dev. coeff. DWT—delta	2.825	0.275	3.362	0.290	2.507	0.549	0.810
*x*_12_	St. dev. coeff. DWT—theta	1.795	0.180	1.709	0.366	2.181	0.505	0.300
*x*_13_	St. dev. coeff. DWT—alpha	1.266	0.140	0.766	0.175	1.275	0.288	1.276
*x*_14_	St. dev. coeff. DWT—beta	0.556	0.122	0.267	0.072	0.466	0.146	1.057
*x*_15_	St. dev. coeff. DWT—gamma	0.154	0.039	0.085	0.028	0.115	0.040	0.596
*x*_16_	Relative power DWÒ—delta	0.501	0.097	0.708	0.118	0.408	0.175	0.873
*x*_17_	Relative power DWÒ—theta	0.203	0.039	0.190	0.081	0.311	0.132	0.347
*x*_18_	Relative power DWÒ—alpha	0.202	0.043	0.077	0.035	0.213	0.097	0.913
*x*_19_	Relative power DWÒ—beta	0.081	0.038	0.020	0.011	0.060	0.039	0.613
*x*_20_	Relative power DWÒ—gamma	0.013	0.007	0.005	0.003	0.008	0.006	0.291
*x*_21_	Correlation dimension—delta	6.979	3.443	6.494	1.605	5.763	1.489	0.045
*x*_22_	Correlation dimension—theta	4.621	0.594	4.288	0.925	4.206	0.884	0.048
*x*_23_	Correlation dimension—alpha	4.184	0.442	3.701	0.886	3.230	0.833	0.272
*x*_24_	Correlation dimension—beta	3.635	0.359	3.097	0.940	2.348	0.832	0.490
*x*_25_	Correlation dimension—gamma	6.729	1.248	6.374	1.838	4.003	1.994	0.493
*x*_26_	Largest Lyapunov exp.—delta	3.282	0.873	2.910	0.856	4.203	1.102	0.327
*x*_27_	Largest Lyapunov exp.—theta	8.213	1.935	8.188	1.914	8.286	1.933	0.000
*x*_28_	Largest Lyapunov exp.—alpha	17.58	2.165	17.57	2.160	17.58	2.377	0.000
*x*_29_	Largest Lyapunov exp.—beta	32.91	5.991	32.65	5.977	33.04	5.091	0.001
*x*_30_	Largest Lyapunov exp.—gamma	11.71	2.985	11.62	2.965	11.89	5.210	0.001

The total variation is the only one feature that we extracted in the time domain. In Table 1, it can be noticed that the total variation has a certain potential for the detection of epileptiform activity in EEG signals. However, the total variation is not that much reliable despite the fact that is a pretty well estimated having in mind the duration of each of the analyzed segments.

**Figure 4:**
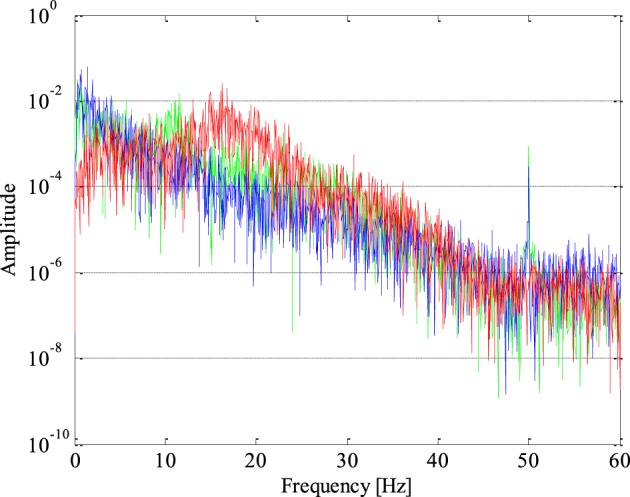
**Periodogram of epileptic (in red) and non-epileptic (unhealthy in blue and healthy tissue in green) segments of EEG signals where a shift in the EEG signal power from lower to higher frequencies in the presence of epileptiform activity is evident**.

The periodogram represents a very important feature of the signal in the frequency domain given that based on it we can get a relative contribution of either any individual frequency or a specific frequency band to the total power of the analyzed signal. The periodograms of one epileptic and two non-epileptic (from both unhealthy and healthy tissue) segments of the EEG signals are shown in Figure 4 where it can be noticed that the EEG signal power of is shifting from lower to higher frequencies in the presence of epileptiform activity.

**Figure 5:**
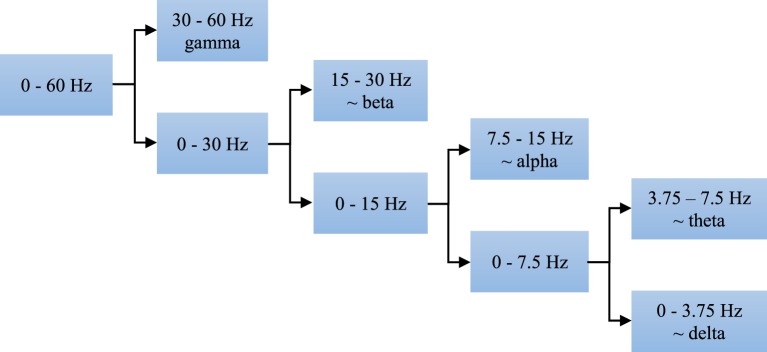
**Four-level decomposition of EEG signal that corresponds to five sub-bands of clinical interest which have better discriminatory characteristics compared with the entire frequency band of 0–60 Hz**.

Using the discrete wavelet transform (DWT) we can completely and independently extract higher and lower frequencies from the signal. All that can be done with different resolution in the time domain, i.e., higher resolution in the time domain for higher frequencies and lower resolution in the time domain for lower frequencies. The EEG signal segments were analyzed at four levels, i.e., the discrete wavelet decomposition was performed at four levels as presented in Figure 5. At the first level of decomposition, the original frequency band of the EEG signals (0–60 Hz) was divided into its higher (30–60 Hz) and lower part (0–30 Hz), i.e., the details and the approximation of the signals at the first decomposition level, respectively. Then at the second decomposition level, the frequency band of the approximation from the first level was additionally divided into its higher (15–30 Hz) and lower (0–15 Hz) part, i.e., the details and the approximation of the signals at the second decomposition level, respectively. After all four decomposition levels, the original band was divided into its five sub-bands, i.e., four sub-bands with the details and one sub-band with the approximation. All these five sub-bands approximately correspond to the earlier defined clinical sub-bands. Power distribution of the EEG signals in the time-frequency domain is quite well described by the DWT coefficients. However, in order to reduce the dimension of the problem and make easier further classification we calculated certain statistics of these coefficients in each sub-band such as the standard deviation and the average relative power, i.e., the square of the absolute values of the DWT coefficients.

Given that the EEG signal also roughly represents a dynamics of a very complex non-linear system such as the brain, the non-linear analysis based on the chaos theory was used in order to extract the information that could not been extracted by any of previously described linear techniques. It is interesting to see that unlike the other feature extraction techniques in the field, a complete agreement about if at all and how to perform a non-linear analysis of the EEG signals has not been achieved yet. Thus, quite often it is possible to find contradictory results of such experiments in the literature. For example, the correlation dimension and the largest Lyapunov exponent have completely different values in Hively et al. (1999), Adeli and Ghosh-Dastidar (2010) and Iasemidis and Sackellares (1991). The feature extraction techniques and non-linear analysis implemented and used in this research are exclusively based on the chaos theory described in the methods part. In addition, there are no any further subjective adjustments applied to the EEG signals, which provides a high level of reproductivity of the obtained results at any time.

**Figure 6:**
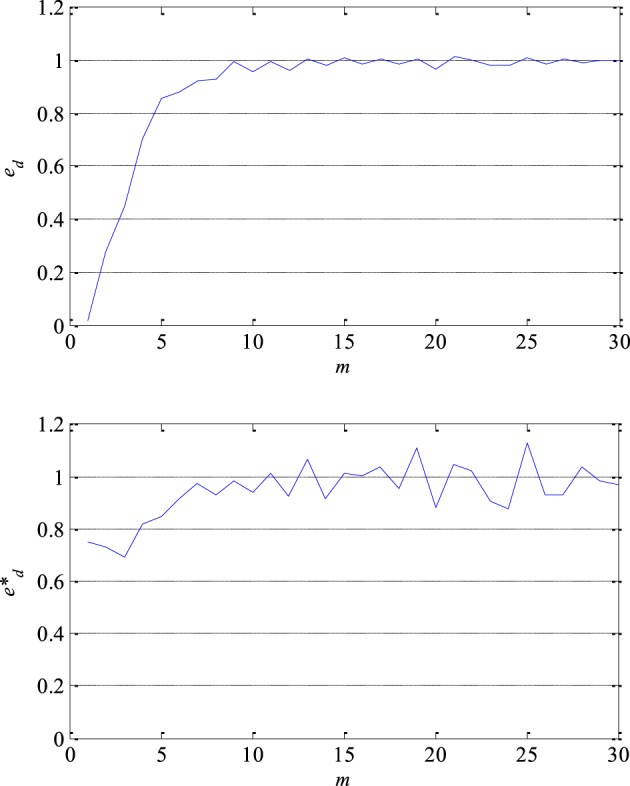
Embedding function *e_d_* (upper) which approaches 1 and thus confirms a presence of a certain level of chaos in EEG signals and redefined embedding function e^*^_d_ (lower) which is not constant for all values of the embedding dimension *m* confirming that chaos is not random but deterministic.

At first, the optimal lag and the embedding dimension were determined in order to reconstruct a segment of the EEG signals in its own lagged phase space. The optimal lag *m_o_* was obtained as the first local minimum of the function of the mutual information coefficients. The value of the optimal lag of the most of analyzed segments varied between 5 and 7. The minimum embedding dimension *d_min_* was determined using Cao's technique, i.e., based on the saturation of the embedding function *e_d_*, for example as presented in Figure 6 in the case of one segment. In other words when a further increase in the embedding dimension does not result in more than 5% of increase in the embedding function. The value of the embedding function of all 300 segments processed approached 1. In fact, this confirms that there is a certain level of chaos present in the segments of the EEG signals. That chaos is not random but deterministic given that the value of the redefined embedding function e^*^_d_ is not constant for all values of the embedding dimension as it can be seen in Figure 6. The value of the minimum embedding dimension varied between 4 and 10.

After reconstruction of the EEG signals in the lagged phase space, the correlation dimension of attractor was estimated using the Taken's estimator. After a few tests the value of radius ε in the phase space was set to 5% of the total size of the attractor since the higher values resulted into to many points, and the smaller ones into insufficient number of points for a good estimation of the correlation dimension. From Table 1, it can be concluded that the correlation dimension as a non-linear feature has a potential for detection of epileptiform activity in EEG signals. It is also obvious that the attractor complexity, i.e., the chaotic behavior of the EEG signals, is lower in presence of epileptiform activity. The values of the correlation dimension in all cases were higher than the embedding dimension of the lagged phase space, which is in accordance with the chaos theory.

**Figure 7:**
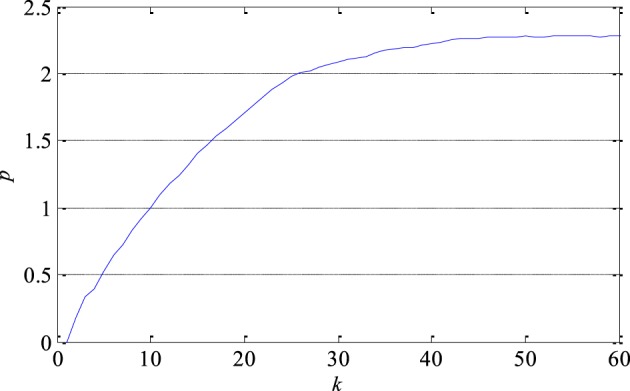
**Prediction error *p* of one segment of EEG signal as a function of the number of samples *k***. Its slope in the middle part determines the largest Lyapunov exponent as a measure of the exponential divergence of nearby phase space trajectories.

The largest Lyapunov exponent as a measure of signal predictability was estimated using Sato's technique. At first, the prediction error as a function of number of samples *k* was determined as shown in Figure 7 in the case of one segment. Then, the largest Lyapunov exponent was estimated based on the function's slope in its medium part. As it can be seen in Table 1, the largest Lyapunov exponent has smaller discrimination ability compared with the correlation dimension. Additionally, it can be also noticed that the presence of epileptiform activity reduces the predictability of the EEG signals since the largest Lyapunov exponent is slightly higher in that case.

### Dimension reduction in feature space

After the feature extraction from all the segments of the EEG signals, obviously none of the individually extracted features is sufficiently reliable for detection of epileptiform activity in EEG signals. This fact represents the main reason to perform the feature extraction in a few different domains of interest, i.e., time, frequency, time-frequency domain and non-linear analysis. The assumption is that the each of them contains some new information about the EEG signal, i.e., the information which is not present in any other domain and thus later contributes to more accurate classification and detection. Therefore, a better separability between the classes of epileptic and non-epileptic segments is expected after an optimal combination of the features from different domains than in the case of using only features from one domain as it is the case with almost all the literature in the field.

Both the KL expansion technique and the dimension reduction technique based on the scatter matrices were tested on the features from all the domains. The obtained results, i.e., adequate separability indexes before and after the dimension reduction in the feature space are presented in Table 2. The reduction technique based on the scatter matrices gives better results in all the domains of interest and also results into the separability index that is, as expected, greater than any individual separability index given in Table 1.

**Table 2:** **Separability indexes after application of two different techniques for dimension reduction in feature space**.

**Features analyzed**	**Dimension**		**Separability index**	
	**Before**	**After**	**KL expansion**	**By the scatter matrices**
Time domain (*x*_1−5_)	5	2	1.93	2.13
Frequency domain (*x*_6−10_)	5	2	1.25	2.16
Time-frequency domain (*x*_11−15_)	10	2	1.40	4.78
Non-linear analysis (*x*_16−20_)	10	2	1.07	1.15

In Table 2, one can see that out of all the analyzed features, the highest separability index and the best discrimination characteristics between epileptic and non-epileptic segments have the features obtained in time-frequency domain after the DWT. However, the other features despite their lower separability indexes are also useful for later classification that is concluded based on an additional analysis whose results are presented in Table 3. It can be noticed that starting from the features in time domain the separability index increases by a gradual inclusion of the features from other domains.

**Table 3:** **Separability indexes after the reduction based on the scatter matrices and gradual involvement of features from different domains**.

**Features analyzed**	**Dimension**		**Separability index**
	**Before**	**After**	
Time domain (*x*_1−5_)	5	2	2.13
Including frequency domain (*x*_1-10_)	10	2	3.52
Including time-frequency domain (*x*_1−20_)	20	2	6.74
Including non-linear analysis (*x*_1−30_)	30	2	8.78

**Figure 8:**
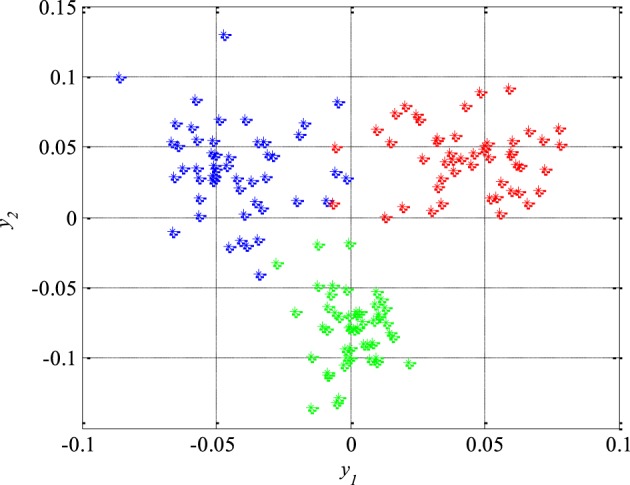
**Epileptic (in red) and non-epileptic (unhealthy in blue and healthy tissue in green) EEG signals in a new two-dimensional feature space after dimension reduction based on scatter matrices**.

Unlike the previous figures, Figure 8 shows 50 original nineteen-dimensional feature vectors *X*, which correspond to 50 segments from each of the three classes of the EEG signals, mapped into their new reduced two-dimensional feature space. All these 150 two-dimensional vectors *Y* will be later used in the next section for the design of appropriate classifiers while the rest of 150 segments and their corresponding feature vectors will be used to test the performance of the designed classifiers as well as the total accuracy of the new technique for detection of epileptiform activity in EEG signals.

### Classification

After the reduction of the feature space dimension to two, the next step is the design of appropriate classifiers that can separate epileptic from non-epileptic segments of the EEG signals in the reduced feature space shown in Figure 8. This represents the last step in design of the new technique for detection of epileptiform activity in EEG signals. Having in mind the nature of the EEG signals and possible changes in their statistical properties it is very desired to use robust classifiers. Based on Figure 8 it can be concluded that quadratic classifiers represent quite logical choice for classification even though these three classes of the EEG signals are also piecewise linearly separable but with a much higher classification error. In total two quadratic classifiers were designed following the procedure described in Section Design of Quadratic Classifiers.

**Figure 9:**
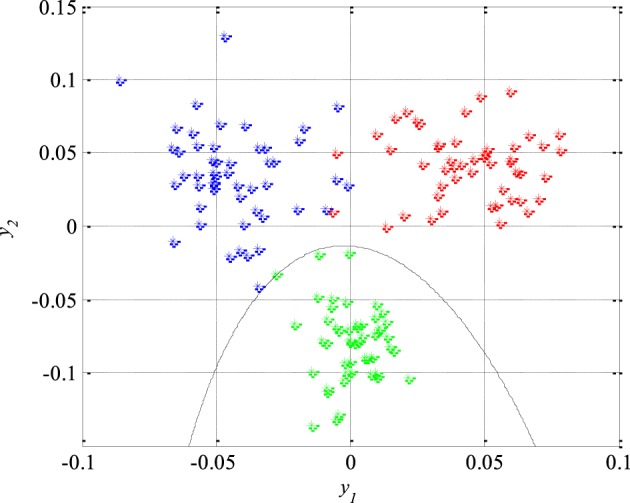
**The first quadratic classifier which separates non-epileptic EEG signals of healthy tissue (in green) from non-epileptic (in blue) and epileptic EEG signals of unhealthy tissue (in red) during the design and training phase**.

**Figure 10:**
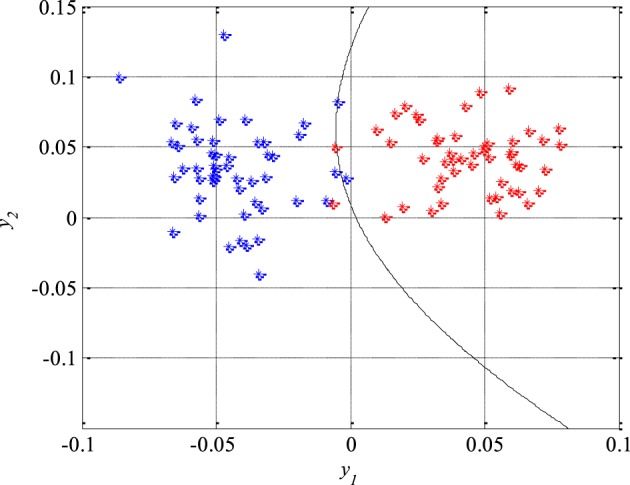
**The second quadratic classifier which separates epileptic (in red) from non-epileptic EEG signals of unhealthy tissue (in blue) during the design and training phase**.

As it can be seen in Figure 9, the first classifier separates the non-epileptic segments of the EEG signals of healthy brain tissue (in green) from the non-epileptic segments of unhealthy tissue (in blue) as well as from the epileptic segments (in red). This classifier is defined using the following equation:

(44) $$h(Y)=\sum_{i=1}^{2}\sum_{j=1}^{2}q_{ij}y_{i}y_{j}+\sum_{i=1}^{2}\nu_{i}y_{i}+\nu_{0}$$

where the unknown parameters are *q*_11_ = −4870.8, *q*_12_ = *q*_21_ = −239.9, *q*_22_ = −174.9, ν_1_ = −29.2, ν_2_ = −174.9 and ν_0_ = −2.3. After that, the second classifier which separates the remaining two unseparated classes of the EEG signals segments, i.e., the epileptic and the non-epileptic segments of unhealthy brain tissue, was designed. The parameters of the Equation (44) for this classifier are *q*_11_ = −436.7, *q*_12_ = *q*_21_ = −128.2, *q*_22_ = 444.6, ν_1_ = −237.9, ν_2_ = −57.2 and ν_0_ = 0.5 while the classifier itself is shown in Figure 10.

**Figure 11:**
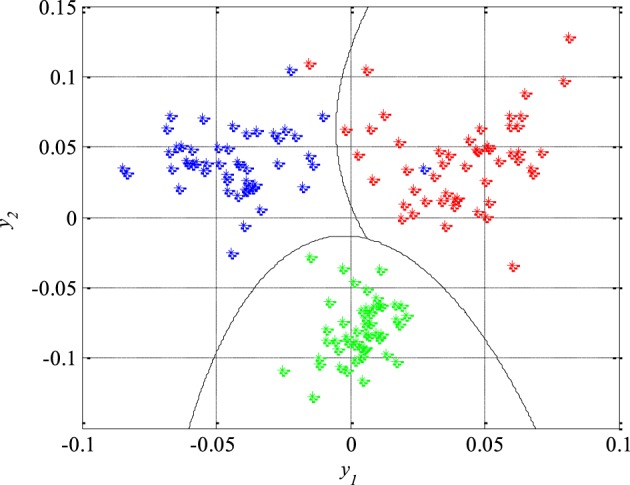
**Piecewise quadratic classifier which separates epileptic (in red) from non-epileptic (unhealthy in blue and healthy in green) EEG signals of the test set**.

The performance of the designed classifiers and thus the new technique for detection of epileptiform activity in EEG signals was tested by classification of the remaining 150 segments which were not previously used during the design procedure. The obtained results are presented in Figure 11, where the piecewise quadratic classifier is just a combination of two quadratic classifiers.

The classification results can also be represented by a confusion matrix that is given in Table 4, where its each cell contains number of classified features for each combination of three classes of the EEG signals segments. Based on the confusion matrix and Figure 11, it can be concluded that all the non-epileptic segments of healthy tissue were correctly classified. However, the remaining two classes contained one segment each which was incorrectly classified, i.e., classified as it belongs to the other class. The statistical performances such as sensitivity, specificity and accuracy, of the designed piecewise quadratic classifiers are presented in Table 5. As it can be seen, the total accuracy of the new technique for detection of epileptiform activity in EEG signals is 98.7%. Typically, quadratic classifiers are robust and do not exhibit overtraining when the number of parameters to be estimated is much less than the number of samples as in this case. Anyway, it is a good practice to cross validate this piecewise classifier in order to ensure its stability. A five-fold cross validation was performed and it resulted in the cross-validation loss, i.e., the error of the out-of-fold samples, of 1.7%. Even though it is slightly higher than the classification error of 1.3% it gives a confidence that the classifier is reasonably accurate.

**Table 4:** **Confusion matrix**.

**EEG signals (input/output)**	**Non-epileptic**		**Epileptic**
	**Healthy**	**Unhealthy**	
Non-epileptic of healthy brain tissue	50	0	0
Non-epileptic of unhealthy brain tissue	0	49	1
Epileptic	0	1	49

**Table 5:** **Statistical performances**.

**EEG signals**	**Statistical performances [%]**		
	**Sensitivity**	**Specificity**	**Accuracy**
Non-epileptic of healthy brain tissue	100	100	98.7
Non-epileptic of unhealthy brain tissue	98	99	
Epileptic	98	99	

## Discussion

Having in mind the results of other techniques available in the literature, presented in Table 6 and tested on the identical segments of the EEG signals, the new technique demonstrated a very good performance. The accuracy of the other techniques varied between 85 and 99%. In addition to high accuracy achieved, it should also be emphasized that all the segments of the analyzed EEG signals were normalized before the feature extraction. In that way we managed to overcome one of the main disadvantages of the techniques from Table 6 in terms of real clinical application, i.e., those techniques rely on the amplitude of the EEG signals as one of the key discriminatory features. However, the EEG signal amplitude has been found as unreliable in real clinical applications since it varies significantly even with healthy individuals, depending on other brain activities as well as other activities of human body. Also, some other undesired effects, e.g., different electrodes used for recording, different patients and their brain tissues, on the detection technique has also been removed by normalization. Unlike the techniques from Table 6, which are mainly based only on features from one of the domains, the new technique relies on carefully extracted features from all the domains of interest including non-linear analysis as well. Because of that, this technique is more robust and less sensitive on changes in the EEG signals that dominantly impact the features from one or two domains while at the same time are invisible in other domains and do not have any relation with a presence of epileptiform activity in EEG signals to be detected.

**Table 6:** **Other techniques for detection of epileptic EEG signals**.

**Authors and year**	**Feature extraction**	**Classification**	**Accuracy**
Nigam and Graupe, 2004	Non-linear filter	Diagnostic neural networks	97.2
Kannathal et al., 2005a	Non-linear analysis	Surrogate data analysis	90.0
Kannathal et al., 2005b	Entropy	Adaptive neuro-fuzzy inference system	92.2
Guler and Ubeyli, 2005	Lyapunov exponents	Recurrent neural networks	96.8
Ubeyli, 2006	Lyapunov exponents	Artificial neural networks	95.0
Sadati et al., 2006	Wavelet transform	Adaptive neuro-fuzzy network	85.9
Subasi, 2007b	Wavelet transform	Expert models	95.0
Tzallas et al., 2007	Time-frequency domain analysis	Artificial neural networks	99.3
Chua et al., 2008	Power spectral density	Gaussian mixture model	93.1
Ghosh-Dastidar et al., 2008	Principal component analysis	Artificial neural networks	99.3
Ocak, 2008	Wavelet transform, approximate entropy and genetic algorithm	Learning vector quantization	98.0
Mousavi et al., 2008	Wavelet transform and autoregressive model	Artificial neural networks	96.0
Ubeyli, 2008	Wavelet transform	Expert models	93.2
Chandaka et al., 2009	Crosscorrelation	Support vectro machines	96.0
Ocak, 2009	Wavelet transform and approximate entropy	Surrogate data analysis	96.7
Guo et al., 2009	Wavelet transform and relative wavelet energy	Artificial neural networks	95.2
Naghsh-Nilchi and Aghashahi, 2010	Eigenvector methods	Artificial neural networks	97.5
Guo et al., 2011	Genetic programming	*K*-nearest neighbor classifier	93.5
Orhan et al., 2011	Wavelet transform	Cauterization and artificial neural networks	96.7
Gajić et al., 2014	Wavelet transform and dimension reduction based on scatter matrices	Quadratic classifiers	99.0

In order to further increase the detection accuracy of the new technique during its real clinical application, a previous elimination of artifacts is very desirable immediately after acquisition of the EEG signals, i.e., before any further processing and feature extraction. The artifacts removal can be performed very reliably using some of already developed and available techniques (Hyvarinen et al., 2001; Rosso et al., 2002). In addition, it is also necessary to make a certain compromise in terms of duration of the segments to be sequentially analyzed in real time. The segment duration should be subsequently adjusted depending on both application and patient. Not only during the feature extraction and the dimension reduction in the feature space, but also during the design of classifiers, a special attention has been paid to the robustness of the detection technique. This resulted in the choice of quadratic classifiers which in addition to their simplicity are known for a high level of robustness in the applications of this type. Quadratic classifiers have also one more important feature that is possibility of visualization of the classification results in two-dimensional space. Namely, despite the fact that the mapped features *y*_1_ and *y*_2_ as a linear combination of the original features *x_i_* extracted from the different domains cannot be anymore associated to certain properties of the EEG signals, they still can provide some further useful insights. For example, in Figure 11 it can be noticed that the feature *y*_1_ can help during determination of the damage level of the brain tissue, while the feature *y*_2_ indicates either presence or absence of epileptic EEG signal.

As part of our future work we plan an additional testing on other bigger and mainly commercially available data bases of the EEG signals (e.g., http://epilepsy-database.eu) containing much more interictal, preictal and ictal EEG data with the aim of further development and adaptation of the new technique for use in a real clinical environment. We will also try to access its potential in the field of emotion detection (e.g., happiness, sadness, depression, alertness, etc.) as well as detection of abnormal activities associated with some other brain disorders such as Alzheimer's disease and schizophrenia.

### Conflict of interest statement

The authors declare that the research was conducted in the absence of any commercial or financial relationships that could be construed as a potential conflict of interest.
